# Predictors of modern contraceptive use during the postpartum period among women in Uganda: a population-based cross sectional study

**DOI:** 10.1186/s12889-015-1611-y

**Published:** 2015-03-18

**Authors:** Gideon Rutaremwa, Allen Kabagenyi, Stephen Ojiambo Wandera, Tapiwa Jhamba, Edith Akiror, Hellen Laetitia Nviiri

**Affiliations:** Centre for Population and Applied Statistics (CPAS), Makerere University, Kampala, Uganda; United Nations Population Fund (UNFPA), Uganda Country Office, Kampala, Uganda; Uganda Bureau of Statistics (UBOS), Ministry of Finance and Planning, Kampala, Uganda

**Keywords:** Predictors, Postpartum, Family Planning, Contraception, Women, Uganda

## Abstract

**Background:**

The rationale for promotion of family planning (FP) to delay conception after a recent birth is a best practice that can lead to optimal maternal and child health outcomes. Uptake of postpartum family planning (PPFP) remains low in sub-Saharan Africa. However, little is known about how pregnant women arrive at their decisions to adopt PPFP.

**Methods:**

We used 3298 women of reproductive ages 15–49 from the 2011 UDHS dataset, who had a birth in the 5 years preceding the survey. We then applied both descriptive analyses comprising Pearson’s chi-square test and later a binary logistic regression model to analyze the relative contribution of the various predictors of uptake of modern contraceptives during the postpartum period.

**Results:**

More than a quarter (28%) of the women used modern family planning during the postpartum period in Uganda. PPFP was significantly associated with primary or higher education (OR=1.96; 95% CI=1.43-2.68; OR=2.73; 95% CI=1.88-3.97 respectively); richest wealth status (OR=2.64; 95% CI=1.81-3.86); protestant religion (OR=1.27; 95% CI=1.05-1.54) and age of woman (OR=0.97, 95% CI=0.95-0.99). In addition, PPFP was associated with number of surviving children (OR=1.09; 95 % CI=1.03-1.16); exposure to media (OR=1.30; 95% CI=1.05-1.61); skilled birth attendance (OR=1.39; 95% CI=1.12-1.17); and 1–2 days timing of post-delivery care (OR=1.68; 95% CI=1.14-2.47).

**Conclusions:**

Increasing reproductive health education and information among postpartum women especially those who are disadvantaged, those with no education and the poor would significantly improve PPFP in Uganda.

## Background

The rationale for promotion of family planning (FP) to delay conception after a recent birth is a best practice that can lead to optimal maternal and child health outcomes. On the contrary, short inter-pregnancy intervals can result in negative health outcomes such as maternal anaemia, low birth weight, and neonatal/infant mortality [1,2].

The postpartum period is a time when many routine interventions are provided to mothers [3]. Uptake of postpartum family planning (PPFP) remains low in sub-Saharan Africa and very little is known about how pregnant women arrive at their decisions to adopt PPFP [4]. Yet, the benefit of early adoption or continuation of family planning are well known and are positive [5-7].

Postpartum months are a challenging time for women because of breastfeeding, childcare, menstrual resumption, and resumption of sexual relations. In a study of women residents of two Nairobi settlements of Korogocho and Viwandani, results showed that sexual resumption occurred earlier than that of menstruation and postpartum contraceptive use [2]. Resumption of sex puts woman at the risk of conception and therefore, creates the need for postpartum contraception.

Some evidence is available to support selected postpartum contraceptive methods [3,8-14]. These methods include the Intra Uterine Device (IUD), the progestin-only oral contraception (POC) among others. Some authors have pointed out that an efficient contraceptive during the postpartum period is lactational amenorrhea (LAM), which is highly acceptable by some religious authorities [8].

The choice of a post-partum contraceptive method depends on many factors, including the need for a temporary versus a permanent method, the infant feeding choice and the extent to which informed consent is made prior to delivery [12]. One of the reasons put forward for limited use of some hormonal contraceptive methods during the postpartum period is often their effects on lactation and infant growth [15]. In addition, other side effects such as heavy menstrual bleeding and pain, have been associated with these methods [16].

Although concerns remain that using a progestin contraceptive in the early postpartum period could negatively affect lactogenesis, as well as the quantity and quality of the breast milk [12], the postpartum contraceptive options available to mothers include progestin-only oral contraceptives; intrauterine devices, including the levonorgestrel intrauterine contraceptive; barrier methods; and the etonogestrel implant [6,11]. Earlier research evidence [17] suggests that postpartum and intrapartum application of the IUD technique is feasible and does not interfere with normal uterine involution. Moreover, the ability to utilize the IUD in the immediate postpartum period, when motivation for acceptance of birth control devices is at its peak, provides a clinician with another tool for use in women who are unlikely to return for follow-up visits [11,16,18].

High rates of acceptance of contraceptive services in the immediate postpartum period indicate that this period is the most effective means of initially reaching the women in need of family planning services [19]. In the United States, the National Surveys of Family Growth data showed that as of 1982, most lactating women who were sexually active used a contraceptive method; barrier methods were most frequently used. However, black women and those of higher parity and lower educational level, were more likely to be sexually active and not using a method [7]. In a related study, Weston, Neustad and Gilliam [20], suggest that the facilitators to IUD uptake included strong recommendations from providers or family members, planning for IUD during pregnancy, and perceived reproductive autonomy. Incidences of repeat pregnancy were reported among participants who did not obtain IUDs and instead used condoms, used no method, or intermittently used hormonal methods in the same study.

Planning for postpartum contraception is particularly important for pregnant women at risk of rapid repeat unintended pregnancy. Yee and Simon in 2012 [5] in their study at a medical center in Chicago, used a qualitative approach to examine women’s perspectives toward the optimal provision of comprehensive contraceptive counseling. The findings suggest that focused antenatal contraceptive counseling about postpartum contraception increased the possibility of use [5].

In the current study, we posit that individual socio-economic and demographic factors operate through societal and institutional factors to increase or diminish the demand for PPFP. Invariably, this conceptualization mirrors the basic tenets of other Health-Belief Models - HBM [21-23]. The HBM has been previously applied to studies on family planning use and consistently explains the importance of a number of factors in the use of FP [24,25]. The basic argument in the HBM framework is that, there are factors that influence a person’s decision to use a PPFP. These include: 1) Enabling factors like availability of PPFP services, organization of the health system, economic means (income, insurance, transport etc.); 2) Predisposing characteristics (Demographic factors, social structures and beliefs); and 3) Need (perceived and evaluated). In this study, we attempt to analyze some of these factors as indicated in the methods section.

Therefore, the key question addressed by this study is, what are the predictors of modern contraceptive use during the post-partum period in Uganda? The study therefore, addressed the following two specific objectives. First, it sought to establish the level of contraceptive use during the postpartum period; and second, was to analyze the predictors of PPFP in Uganda.

## Methods

### Data source and study sample

We used secondary population-based cross-sectional survey data – the Uganda Demographic and Health Survey (UDHS) conducted in 2011. Although these data are already in public domain, authorization to use the data was obtained from MEASURE DHS by providing a brief description of the study through their website.

The UDHS employed nationally representative sample, which were based on a two-stage stratified sample of households. These types of surveys are carried out every after five years to provide information on national demographic, socio-economic, health indicators including contraceptive use among women. These surveys are conducted by the Uganda Bureau of Statistics (UBOS) in collaboration Macro International.

A total of 8,674 women aged 15–49 was the sample for the 2011 DHS. Informed consent for participation in the study was acquired from all the respondents. Detailed information about data collection procedures including the informed consent form are contained in the UDHS 2011 report [26].

A total of 3298 postpartum women aged 15–49 years were extracted from the 2011 UDHS data for further analyses. Women included in the analysis, had completed the full 12 months of the postpartum period. Only the most recent birth was considered, even if a woman delivered more than once during the reference period. We excluded women who did not have a live birth in the five years before the survey.

### Variables

We defined postpartum contraceptive or post-partum family planning (PPFP) as a woman’s use of any modern method of contraception during the 12 months following her most recent childbirth. The modern methods included pill, IUD, injection, diaphragm, condom (male or female), sterilization (male or female), implant, or foam/jelly). Therefore, the outcome variable in this study – PPFP, was expressed as the binary outcome, whether a woman utilized modern postpartum family planning or otherwise. PPFP was coded 1, if a woman used any modern method of contraception and coded 0 otherwise.

Predictor variables considered (see Table 1) included those commonly reported in the contraceptive use studies. They included (categories are given in parentheses) : place of residence (urban, rural), education level attainment (no education, primary, secondary and higher), religion (Catholic, Protestant, Muslims, others; where other religions include Seventh Day Adventists (SDA) and unknown religions), wealth index (poorest, poor middle, rich and richest), region (Kampala, Central, Eastern, Northern and Western), marital status (never married, currently married, previously married). Additional variables included the following: age of the woman (continuous), number of surviving children, exposure to media (whether a woman had ever accessed family planning information through print, radio and television channels), skilled birth attendance and timing of post-delivery check-up. The UBOS generated these variables and their coding categories before availing them for public use.

**Table 1:** **Percentage distribution of postpartum women age 15–49 by selected characteristics**

**Variable**	**Category**	**Frequency**	**Percent (%)**
**Education level attainment**	None	509	15.4
	Primary	2006	60.8
	Secondary +	783	23.7
**Region of residence**	Kampala	252	7.6
	Central	655	19.9
	Eastern	867	26.3
	Northern	638	19.3
	Western	886	26.9
**Religion**	Catholic	1,351	41.0
	Protestant	1,374	41.7
	Muslim	452	13.7
	Other	121	3.7
**Mean Age of women**	(Continuous)	3298	29.7
**Residence**	Urban	544	16.5
	Rural	2753	83.5
**Marital status**	Never married	112	3.4
	Married	2754	83.6
	Previously married	430	13.0
**Wealth Index**	Poorest	684	20.7
	Poorer	675	20.5
	Middle	617	18.7
	Richer	593	18.0
	Richest	729	22.1
**Mean number of living children**	(Continuous)	3298	3.9
**Exposure to media**	No	855	25.9
	Yes	2443	74.1
**Timing of post-delivery checkup**	None	2030	61.6
	<1 day	1021	31.0
	1-2 days	136	4.1
	3+ days	110	3.3
**Skilled birth attendant**	Not skilled	1032	31.3
	Skilled	2036	61.7
	TBA	230	7.0
**Used PPFP**	No	2384	72.3
	Yes	914	27.7
**Total**		**3298**	**100.0**

### Data analysis

Descriptive statistics (either as percentages for the categorical variables or mean for the continuous variables) are shown for selected predictors (Table 1). Bivariate analyses (based on Pearson chi-square tests) were performed to examine the association between PPFP and each of the selected predictors (Table 2). The chi-square tests were used to analyze differences in all variables between the two groups. All predictors with significant difference (p < 0.05) were selected and included in a multivariate logistic regression analysis.

**Table 2:** **Percent Distribution of women aged 15–49 by use of PPFP and selected background characteristics**

**Variable**	**Category**	**Non-use (%)**	**Used PPFP (%)**	**Frequency**	**P-value**
**Education level attainment**					
	None	88.1	11.9	509	
	Primary	74.0	26.0	2006	0.000
	Secondary+	57.6	42.4	783	
**Region of residence**					
	Kampala	54.8	45.2	252	
	Central	63.8	36.2	655	0.000
	Eastern	72.8	27.3	867	
	Northern	83.0	17.0	638	
	Western	75.4	24.6	886	
**Religion Catholic**					
	Catholic	75.8	24.2	1,351	
	Protestant	71.5	28.5	1,374	
	Muslim	63.5	36.5	452	0.000
	Other	74.5	25.5	121	
**Age-group**					
	15-19	75.9	24.1	162	
	20-24	69.4	30.6	709	
	25-29	67.8	32.2	872	0.000
	30-34	72.5	27.5	610	
	35-39	75.2	24.8	544	
	40-44	77.2	22.8	302	
	45-49	94.2	5.9	99	
**Residence Urban**					
	Urban	56.3	43.7	544	
	Rural	75.5	24.6	2753	0.000
**Marital status**					
	Never married	67.2	32.8	112	
	Married	71.6	28.4	2754	0.206
	Previously married	79.1	20.9	430	
**Wealth Index**					
	Poorest	88.9	11.1	684	
	Poorer	79.5	20.5	675	
	Middle	74.4	25.6	617	0.00
	Richer	69.0	31.0	593	
	Richest	57.3	42.7	729	
**Number of living children**					
	<3	69.4	30.6	1110	
	3-5	71.7	28.3	1338	0.002
	6+	77.0	23.0	849	
**Exposure to media**					
	No	81.3	18.7	855	
	Yes	69.2	30.9	2443	0.000
**Timing of post-delivery care checkup**					
	Received none	75.9	24.1	2030	
	<1 day	67.5	32.5	1021	
	1-2 days	68.5	31.5	136	0.000
	3+ days	55.8	44.2	110	
**Skilled birth attendant**					
	Not skilled	81.3	18.7	1032	
	Skilled	66.6	33.4	2036	0.000
	TBA	82.2	17.8	230	

Finally, odd ratios (ORs) based on the 95% confidence intervals were calculated for each group of the categorical predictors (Table 3). The logistic regression models were used to predict the log-odds of using modern contraceptives during the postpartum period.

**Table 3:** **Logistic regression model predicting the log-odds of utilizing PPFP controlling for selected variables (Adjusted odds-ratios are presented)**

**Variable/category**	**Model (1)**		**Model (2)**		**Model (3)**	
	**Odds-ratio**	**95% CI**	**Odds-ratio**	**95% CI**	**Odds-ratio**	**95% CI**
**Education level attainment**						
None (Rc)			1.000	-	1.000	-
Primary			**2.099	[1.537-2.868]	**1.957	[1.429-2.682]
Secondary +			**3.027	[2.094-4.376]	**2.730	[1.879-3.968]
**Region of residence**						
Kampala (Rc)			1.000	-	1.000	-
Central			1.351	[0.967-1.889]	1.389	[0.990-1.948]
Eastern			1.195	[0.838-1.704]	1.217	[0.848-1.746]
Northern			0.900	[0.631-1.309]	0.866	[0.597-1.256]
Western			1.051	[0.734-1.505]	1.094	[0.759-1.576]
**Wealth Index**						
Poorest (Rc)			1.000	-	1.000	-
Poorer			**1.564	[1.143-2.139]	**1.442	[1.052-1.975]
Middle			**1.911	[1.384-2.639]	**1.692	[1.218-2.350]
Richer			**2.249	[1.614-3.133]	**1.993	[1.426-2.784]
Richest			**3.121	[2.148-4.533]	**2.643	[1.812-3.856]
**Religion**						
Catholic (Rc)			1.000	-	1.000	-
Protestant			*1.262	[1.043-1.526]	**1.271	[1.050-1.539]
Muslim			1.047	[0.810-1.355]	1.059	[0.816-1.375]
Other			0.999	[0.607-1.642]	0.953	[0.586-1.551]
**Age of woman (continuous)**	**0.981	[0.963-0.998]	**0.969	[0.950-0.987]	**0.969	[0.950-0.988]
**Place Residence**						
Urban (Rc)			1.000	-	1.000	-
Rural			0.845	[0.651-1.095]	0.891	[0.686-1.157]
**Number of living children**	1.013	[0.958-1.071]	**1.085	[1.022-1.152]	**1.090	[1.026-1.158]
**Exposure to media**						
No (Rc)	1.000	-			1.000	-
Yes	**1.891	[1.552-2.303]			**1.300	[1.049-1.611]
**Skilled birth attendant**						
No (Rc)	1.000	-			1.000	-
Skilled attendance	**1.943	[1.579-2.390]			**1.393	[1.116-1.738]
TBA	0.956	[0.630-1.449]			0.889	[0.581-1.360]
**Timing of post-delivery care checkup**						
Received None (Rc)	1.000	-			1.000	-
<1 day	0.997	[0.823-1.207]			0.893	[0.728-1.095]
1-2 days	**1.537	[1.068-2.211]			**1.681	[1.144-2.470]
3+ days	**2.090	[1.429-3.056]			**2.141	[1.429-3.207]
**Model constant**	**0.225	[0.145-0.351]	**0.151	[0.081-0.283]	**0.108	[0.057-0.206]

Note: Rc= reference category; ** and bold=significant finding at p<95%.

Formally, the estimated equation may be expressed as follows:

1 $$ \operatorname{logit}\left[P\left(Y=1\right)\right]={\beta}_0+{\displaystyle \sum_{j=1}^k{\beta}_j{X}_j} $$

Where; logit [P(Y=1)] refers to the natural log odds that a respondent will: use postpartum contraception, β_0_ refers to the intercept of the regression model; and β_j_X_j_ refer to regression estimates for the set of explanatory variables (numbered 1 through k) included in the models.

For purposes of accounting for the complex sample design, used in DHS data collection, we weighted the data during the analysis. This procedure was intended to iron out the effects of clustering and stratification as well as the effect of sampling weights when computing the variance, standard error, and confidence intervals (the “*vce – robust*” option was used). The variables were also checked for multi-collinearity, and those variables that passed the test were the ones included in the final model. We analyzed the data using STATA software version 13.

## Results

### Descriptive results

Table 1 presents selected background characteristics for postpartum women – women who had their most recent birth in the period five years prior to the date of interview. More than half of the postpartum women had primary education and lived in eastern and western regions. Nearly half of the women were Catholics. Protestants had similar distribution. The women’s mean age was 30 years. More than three quarters of the women were rural residents and were married. Nearly half of the women were from the poorest and poorer wealth status.

Women had an average of four surviving children. Nearly three quarters of the women had exposure to family planning on the mass media – newspaper, radio or television. The majority of women did not receive any post-delivery check-up. More than half of the women had a skilled birth attendant. Finally, slightly over a quarter of the women received modern postpartum family planning services.

### Association between PPFP and selected predictors

Table 2 shows the percent distribution of women aged 15–49 by their use of modern postpartum family planning. There was a strong association between PPFP use and women’s background characteristics, with the exception of marital status. Consequently, we dropped marital status from the multivariable logistic regression models due to its non-significance.

### Multivariate results

Table 3 shows the log-odds of utilization of modern postpartum family planning by women, controlling for selected predictor variables. In Model 1, we included proximate predictors of PPFP only. In Model 2, we added socio-economic factors. In model 3, we controlled for both proximate and socio-economic factors.

In Model 1, the results show that a one-year increase in age of the woman was significantly associated with a slight reduction in the odds of utilizing PPFP (OR=0.98; 95% CI=0.96-1.00). This finding was consistent even after controlling for socio-economic factors in Models 2 and 3. Furthermore, Models 2 and 3 show that a one-child increment in the number of living children was associated with an increase in the log-odds of using PPFP of about 9 percent (OR=1.09; 95% CI=1.022-1.15).

As expected, women who had exposure to media on family planning were significantly more likely to use PPFP, compared to those who did not. The results shown in Model 1 suggest that women with media exposure had 1.9 times log-odds (OR=1.89; 95% CI=1.55-2.30) of using PPFP compared to those who had no such exposure. Even after controlling for socio-economic factors (Model 3), this relationship persisted (OR=1.30; 95% CI=1.05-1.61).

Women who received skilled birth attendance were more likely to utilize PPFP compared to those who did not deliver (OR=1.94; 95% CI=1.58-2.39). In final model, after controlling for all other factors, this relationship was maintained, although with slight reduction as shown in Model 3 (OR=1.39; 95% CI=1.12-1.74).

The timing of receipt of post-delivery care was associated with PPFP. Longer timing of receipt of post-delivery care was associated with higher likelihood of utilization of PPFP. Women who took 1–2 days to get post-delivery care had 1.5 times the log odds of utilizing PPFP (95% CI=1.07-2.21), compared to those who did not receive post-delivery. This relationship was even stronger after controlling for all other variables in third model (OR=1.68; 95% CI=1.14-2.47).

Women’s education significantly predicted PPFP utilization. The log odds of PPFP use were higher among women primary education in Model 2 (OR=2.09; 95% CI=1.54-2.87) and Model 3 (OR=1.96; 95% CI=1.43-2.68). Similarly, PPFP utilization was increased among women with secondary education in Model 2 (OR=3.03; 95% CI=2.09-4.38) and Model 3 (OR=2.73; 95% CI=1.88-3.97). This relationship was consistent even after controlling for all the variables in Model 3, although with some reduction.

PPFP utilization was significantly associated with higher wealth status. Women in higher wealth quintiles were significantly more likely to use PPFP compared to those within the lowest wealth quintiles. This finding was consistent in both Model 2 and Model 3. Model 2 showed that compared to women in the poorest category, the log-odds of using PPFP increased from 1.5 (95% CI=1.14-2.14) among women in the poorer category to 1.9 (95% CI=1.38-2.64) among the middle wealth index category and were highest among women of richest category (OR=3.12; 95% CI=2.15-4.53).

Finally, religious affiliation was also associated with PPFP. The findings in Models 2 and 3 showed that compared to Catholics, the use of PPFP among Protestants was higher (OR=1.26; 95% CI=1.04-1.53), as shown in Model 2. This latter relationship persisted in the full Model 3 (OR=1.27; 95% CI=1.05-1.54).

## Discussion

This study had two fold objectives: first, estimating the prevalence and second, investigating predictors of modern postpartum family planning use in Uganda. Slightly over a quarter (28%) of the women used modern family planning during the postpartum period. This rate was slightly higher than of all women of reproductive ages in Uganda (26%) in 2011 [26].

Utilization of modern PPF was significantly associated women’s: education level, wealth status, religion, age of the woman, number of surviving children, exposure to the media, and utilization of reproductive health services including skilled delivery care, and timing of post-delivery care.

Our findings show that women’s primary or secondary education predicted utilization of modern PPFP. This relationship is consistent with findings reported by other studies [2,7,27-30]. Higher education level attainment invariably gives postpartum women a better understanding of the available modern contraceptive methods and the benefits of fertility regulation and hence the need for contraception during the postpartum period. In addition, higher education increases awareness of the side effects of contraceptive methods and preference for the most convenient ones [30].

The results indicated a direct relationship between women’s wealth status and the utilization of PPFP. Women from richer households were more likely to use PPFP compared to those in the poorest households. This finding collaborates with a study in Malawi which showed that women in the highest wealth quintiles were more likely to use contraception, relative to those in the poorest income categories [31].

Religious affiliation was generally not significant in the regression models except for the Protestants category. The explanation for this could perhaps lie in the fact that the protestant ethic often tends to be more accepting to contraceptive use compared to Catholics. This argument is consistent with literature elsewhere where Protestant women were more likely than Catholics to use highly effective contraceptive methods [32].

Increment in women’s age significantly reduced their use of PPFP. This implies that PPFP was higher among the younger women compared to the older women. In a study on use of long active reversible contraceptives at a military facility in the US, the study revealed that younger women <25 years were more likely to use these methods [33]. Similar results were also reported concerning current contraceptive use in Malawi, where younger women were more likely to use contraceptives compared to older women [31]. The explanation for this finding is not clear, since all events studied related to the most recent period – five years prior to the survey. In a US study among low income women, use of Depot Medroxyprogesterone Acetate (DMPA) reduced with increasing age of woman [34]. Another significant demographic factor analyzed was the number of living children. The findings showed a direct relationship between a woman’s number of living children and her likelihood of using PPFP.

Women’s exposure to family planning messages on the media increased use of PPFP. Exposure to media content is known to increase demand for services and in the long run, causing behavior change through information, education and communication (IEC) campaigns [18,35,36]. Specific family planning studies in Kenya and Bangladesh have equally reported increase in utilization of family planning methods as a result of exposure to media [37-39]. An earlier study on Uganda using data from the Delivery of Improved Health Services Surveys of 1997 and 1999 also indicated that exposure to Behavioral Change Communication (BCC) messages was associated with increased contraceptive use and intention to use [40].

Furthermore, skilled birth attendance significantly increased utilization of PPFP. This is expected because the motivation to use contraception and discussion with clinicians is usually high in the immediate postpartum period [17]. This study also established that women who had their post-delivery checkup after one day were significantly more likely to use PPFP compared to those who did not have any post-delivery check-up. However, we do not have sufficient explanation for this finding. This finding needs further investigation.

### Study limitations

The cross sectional nature of the data poses causality challenges. It is difficult to ascertain the association between PPFP and the predictor variables since they were measured at one point in time.

The study did not address all health system related factors that affect postpartum family planning utilization. Despite these limitations, we used reliable data and appropriate methods hence the findings reflect accurately on PPFP utilization in Uganda. The large size of this study and its likely representativeness is a great strength.

## Conclusions

From the analysis, the factors that predicted PPFP utilization were women’s primary or higher education level, higher wealth status, protestant religion, younger age, higher number of surviving children, and exposure to family planning through media. In addition, skilled birth attendance and later timing of post-delivery checkup also predicted use of PPFP. These results suggest that any programme aimed at enhancing postpartum contraceptive use should target women with low education, low wealth status, older ages and those with limited exposure to mass media.

Increasing reproductive health education among postpartum disadvantaged and poor women would significantly improve the uptake of PPFP in Uganda. We suggest that the Ugandan government and development partners sponsor family planning messages on radio, in newspapers and television and perhaps even intensify these efforts. Further utilization of alternative strategies like street plays, one-on-one and group counseling in clinics and hospitals would avail information to those who might not have access to print media.

Given the well-documented benefits of postpartum family planning, we recommend that PPFP be fully integrated into maternal health care services. It is likely that such integration would help increase the uptake of modern family planning. This is because delivery and postnatal services remain important windows of opportunity to provide access to family planning messages and to offer women various contraceptive methods.

Finally, there is need for further research on how male involvement and timing of post-delivery check-up influences PPFP in Uganda.

## Abbreviations

BCC: Behavior change communication
IEC: Information education and communication
IUD: Intra uterine device
UDHS: Uganda demographic and health survey
DHS: Demographic and health survey
DMPA: Depot Medroxyprogesterone Acetate
LAM: Lactational Amenorrhea
PPFP: Postpartum family planning

## References

[1] Cleland J, Conde-Agudelo A, Peterson H, Ross J, Tsui AO (2012). Family Planning, Contraception and Health. Lancet.

[2] Ndugwa RP, Cleland J, Madise NJ, Fotso J-C, Zulu EM (2011). Menstrual pattern, sexual behaviors, and contraceptive use among postpartum women in Nairobi urban slums. J Urban Health.

[3] Levitt C, Shaw E, Wong S, Kaczorowski J, Springate R, Sellors J (2004). Systematic review of the literature on postpartum care: selected contraception methods, postpartum Papanicolaou test, and rubella immunization. Birth.

[4] Eliason S, Baiden F, Quansah-Asare G, Graham-Hayfron Y, Bonsu D, Phillips J (2013). Factors influencing the intention of women in rural Ghana to adopt postpartum family planning. Reprod Health.

[5] Yee L, Simon M (2011). Urban minority women’s perceptions and of preferences for postpartum contraceptive counselling. J Midwifery Womens Heal.

[6] Jones K, Egan M, Stevermer JJ (2011). Offer this contraceptive to breastfeeding new moms: Early insertion of the etonogestrel implant does not affect lactogenesis, and fosters contraceptive compliance. J Fam Pract.

[7] Ford K, Labbok M (1987). Contraceptive usage during lactation in the United States: an update. Am J Public Heal J public Heal.

[8] Vekemans M (1997). Postpartum contraception: The lactational amenorrhea method. Eur J Contracept Reprod Heal Care.

[9] Chi I-C, Robbins M, Balogh S (1992). The progestin-only oral contraceptive—its place in postpartum contraception. Adv Contracept.

[10] Tankeyoon M, Dusitsin N, Chalapati S, Koetsawang S, Saibiang S, Sas M (1984). Effects of hormonal contraceptives on milk volume and infant growth: WHO Special Programme of Research and Development and Research Training in Human Reproduction. Contraception.

[11] Edelman DA, Goldsmith A, Shelton JD (1981). Postpartum contraception. Int J Gynecol Obstet.

[12] Kennedy KI (1996). Post-partum contraception. Baillieres Clin Obstet Gynaecol.

[13] Espey E, Ogburn T, Leeman L, Singh R, Ostrom K, Schrader R (2012). Effect of progestin vs. combined oral contraceptive pills on lactation: A double-blind randomized control trial. Obstet s Gnyecology.

[14] Sannisto T, Kosunen E (2009). Initiation of postpartum contraception: a survey among health centre physicians and nurses in Finland. Scand J Prim Health Care.

[15] Brito MB, Ferriani RA, Quintana SM, Yazlle MEHD, Silva de Sá MF, Vieiraa CS (2009). Safety of the etonogestrel-releasing implant during the immediate postpartum period: a pilot study. Contraception.

[16] Backman T (2004). Benefit-risk assessment of the levonorgestrel intrauterine system in contraception. Drug Saf.

[17] Zerzavy FM (1967). Use of intrauterine contraceprive devices in the postpartum period. Am J Public Health.

[18] Udry JR, Clark LT, Chase CL, Levy M (1972). Can mass media advetising increase contraceptive use?. Fam Plann Perspect.

[19] Tamblyn PB, Jacobson J (1970). Continuance of contraception post partum by patients of Cook County Hospital. Public Health Rep.

[20] Weston MRS SLM, Neustad AB, Gilliam ML (2012). Factors influencing uptake of intrauterine devices among postpartum adolescents: A qualitative study. Am J Obstet Gynecol.

[21] Yesudian C (1988). Health Services Utilisation in Urban India.

[22] Rajamohanan KP, Sankey VW, Glick HA, Polsky D, Berlin JA, Lowe RA (2003). Factors affecting Decisions to Seek Treatment for Sick Children in Kerala, India. Soc Sci Med.

[23] Anderson R (1995). Revisiting the behavioural Model and Access to Medical Care: Does it Matter?. J Heal Soc Behav.

[24] Condelli L (1986). Social and attitudinal determinants of contraceptive choice: using the health belief model. J Sex Res.

[25] Speizer IS, Fotso JC, Okigbo C, Faye CM, Seck C (2013). Influence of integrated services on postpartum family planning use: a cross-sectional survey from urban Senegal. BMC Public Health.

[26] UBOS and ICF International Inc. Uganda Demographic and Health Survey. Kampala, Uganda and Calverton - Maryland; 2012:112.

[27] Wanyenze RK, Tumwesigye NM, Kindyomunda R, Beyeza-Kashesya J, Atuyambe L, Kansiime A (2011). Uptake of family planning methods and unplanned pregnancies among HIV-infected individuals: a cross-sectional survey among clients at HIV clinics in Uganda. J Int AIDS Soc.

[28] Creanga AA, Gillespie D, Karklins S, Tsui AO (2011). Low use of contraception among poor women in Africa: an equity issue. Bull World Health Organ.

[29] Lopez LM, Hiller JE, Grimes DA, Chen M (2012). Education for contraceptive use by women after childbirth. Cochrane Database Syst Rev.

[30] Mekonnen W, Worku A (2011). Determinants of low family planning use and high unmet need in Butajira District. South Central Ethiopia Reprod Heal.

[31] Adebowale SA, Adedini SA, Ibisomi LD, Palamuleni ME (2014). Differential effect of wealth quintile on modern contraceptive use and fertility: evidence from Malawian women. BMC Womens Health.

[32] Jones RK, Dreweke J (2011). Countering Conventional Wisdom: New Evidence on Religion and Contraceptive Use.

[33] Dahlke JD, Ramseyer AM, Terpstra ER, Doherty DA, Keeler SM, Magann EF (2012). Postpartum use of long-acting reversible contraception in a military treatment facility. J Women’s Heal.

[34] Dozier AM, Nelson A, Brownell EA, Howard CR, Lawrence RA (2014). Patterns of postpartum depot medroxyprogesterone administration among low-income mothers. J Womens Health (Larchmt).

[35] Wakefield MA, Loken B, Hornik RC (2010). Use of mass media campaigns to change health behaviour. Lancet.

[36] Retherford RD, Mishra V (1997). Media Exposure Increases Contraceptive Use. Natl Fam Health Surv Bull.

[37] Islam MR, Islam MA, Banowary B (2009). Determinants of exposure to mass media family planning messages among indigenous people in Bangladesh: A study on the Garo. J Biosoc Sci.

[38] Cleland J, Bernstein S, Ezeh A, Faundes A, Glasier A, Innis J (2006). Family planning: the unfinished agenda. Lancet.

[39] Westoff CF, Rodriguez G (1995). The mass media and family planning in Kenya. Int Fam Plan Perspect.

[40] Gupta N, Katende C, Bessinger R (2003). Associations of mass media exposure with family planning attitudes and practices in Uganda. Stud Fam Plann.

